# Emerging Role of Biosimilars in Oncology-Hematology in Saudi Arabia: A Practical Perspective

**DOI:** 10.4103/JQSH.JQSH_15_19

**Published:** 2019-12-12

**Authors:** Mansoor A. Khan, Mohammed A. Aseeri, Majed A. Alshamrani, Abdelmajid H. Alnatsheh, Hani S. Alhamdan

**Affiliations:** Department of Pharmaceutical Care, King Abdulaziz Medical City – Western Region (KAMC-WR), Jeddah, Saudi Arabia, King Abdullah International Medical Research Center/King Saud bin Abdulaziz University for Health Sciences, Jeddah, Saudi Arabia

**Keywords:** Biosimilars oncology hematology

## Abstract

Biologics are significant drivers of globally escalating healthcare costs. Biosimilars have potential to offer cost savings with comparable efficacy and safety to innovator products and increase the access of treatment to more patients. This study aimed to increase understanding and perception of biosimilars concept. It also described the pharmacoeconomic impact of biosimilar in oncology and formulary consideration of oncology biosimilars substituting with their originators in major oncology centers in the Saudi Arabia. A biosimilar is a biological product that is similar to a reference biopharmaceutical product. As the manufacturing process hinders the ability to identically replicate the structure of the original product, biosimilar cannot be described as an absolute equivalent of the original medication. Different regulatory agencies such as United States Food and Drug Administration, European Medicines Agency, and Saudi Food and Drug Authority have approved several biosimilars of oncology biologics. The experience of biosimilar use in Europe and USA provides valuable insights into the use of biosimilars. The widespread use of biosimilars has the potential to reduce healthcare expenditure, as well as improving access without compromising patient outcomes. There is a need for increasing awareness about biosimilars to improve acceptance rates. The use of biosimilar filgrastim in Ministry of National Guard Health Affairs, Saudi Arabia, has resulted in a significant cost saving annually. It was proposed that further substitution and switching to biosimilars in oncology would lead to major savings in resources.

## Introduction

Biosimilars are biological medicines that contain a highly similar version of the active substance of an approved biologic reference product. The availability of biosimilars might provide an opportunity to lower healthcare expenditures as a result of the inherent price competition with their reference product. Understanding how biosimilar cancer drugs are regulated, approved, and paid for, as well as their impact in a value-based care environment is essential for physicians and other stakeholders in oncology.[1]

A biosimilar is a biologic product that is similar to a reference biopharmaceutical product. The manufacturing process of biosimilar hinders the ability to identically replicate the structure of the original product, and therefore, it cannot be described as an absolute equivalent of the original medication. The currently available technology does not allow accurate copy of complex molecules, but it does allow the replication of similar molecules with the same activity.[2]

New agents for the treatment and supportive care of cancer have markedly improved therapeutic options and outcome for many malignancies. Biologics include monoclonal antibodies targeted to critical pathways involved in cancer pathogenesis and growth factors to reduce or ameliorate treatment-related hematological toxicity. Unfortunately, access to potentially lifesaving biologics is limited in many areas of the world.[3–5] As the patent expiry of several drugs approaches, there has been intense interest in developing biosimilar agents to introduce cost savings for health-care systems and to widen global access to key biological therapies.[3,4,6] A biosimilar drug is a biological product that is highly similar, but not identical, to a licensed biological product (the reference or originator product).[7–9] Unlike small-molecule generic drugs that are typically chemically synthesized and easy to replicate, it is impossible to make exact copies of reference products because biosimilars (as biologics) are large and highly complex molecules produced in living cells. Structural differences to the reference product may arise because of variations in post-translational modification (such as glycosylation patterns), which could have impact on drug efficacy or safety.[7–9] The development of biosimilars, therefore, involves extensive evaluation and a detailed, comprehensive manufacturing process to ensure that there are no clinically meaningful differences in purity, safety, or potency.[7–9] As is the case for any new therapeutic agent, the evaluation process and approval requirements for a proposed biosimilar may differ between regulatory agencies, leading to differential access based on geographic location.

Extrapolation is the approval of a biosimilar for use in an indication held by the originator biologic not directly studied in a comparative clinical trial with the biosimilar. Extrapolation is a scientific rationale that bridges all the data collected (i.e., totality of the evidence) from one indication for the biosimilar product to all the indications originally approved for the originator.[10] United States (US) Food and Drug Administration (FDA) and the current World Health Organization (WHO) guidance allow the use of clinical efficacy and safety data for one indication to be extrapolated to other indications for the reference biologic. In general, guidelines suggest that extrapolation of data may be allowed for biosimilars as long as sufficient justification can be provided for the new indication (e.g., similar anticipated mechanism of action for the biosimilar) and a rationale for similar pharmacokinetics, efficacy, safety, and immunogenicity can be provided for the new indication target population. This is similar to the existing WHO guidance on extrapolation of clinical data. Examples from the European experience have shown that data for one indication of an innovator may be reasonably extrapolated to another.[11]

Different regulatory agencies such as US FDA, European Medicines Agency (EMA), and Saudi Food and Drug Authority (SFDA) have approved several biosimilars of oncology biologics and more are expected to be approved in near future. As of now EMA has approved 31 biosimilars, US FDA has approved 13 biosimilars and SFDA has approved 4 biosimilars of oncology biologicals. SFDA-listed prices of biosimilars are significantly less than the prices of their originators. It is anticipated that SFDA will approve biosimilar of trastuzumab, rituximab, and bevacizumab in near future. Below mentioned are the lists of the oncology biosimilars approved by EMA [Table 1], US FDA [Table 2], and SFDA [Table 3].[12–14]

**Table 1:** List of EMA-approved oncology biosimilars[12]

**No.**	**Brand Name**	**Active substance**	**Indication**	**Date of approval**
1	Abseamed	Epoetin Alfa	Anemia	August 2007
2	Binocrit	Epoetin Alfa	Anemia	August 2007
3	Epoetin Alfa Hexal	Epoetin Alfa	Anemia	August 2007
4	Retacrit	Epoetin Zeta	Anemia	December 2007
5	Silapo	Epoetin Zeta	Anemia	December 2007
6	Tevagrastim	Filgrastim	Neutropenia	September 2008
7	Filgrastim Hexal	Filgrastim	Neutropenia	February 2009
8	Zarzio	Filgrastim	Neutropenia	February 2009
9	Nivestim	Filgrastim	Neutropenia, HSCT	June 2010
10	Gastrofil	Filgrastim	Neutropenia	October 2013
11	Accofil	Filgrastim	Neutropenia	September 2014
12	Inhixa	Enoxaparin	VTE	September 2016
13	Thorinane	Enoxaparin	VTE	September 2016
14	Truxima	Rituximab	All indications	February 2017
15	Rixathon	Rituximab	All indications	June 2017
16	Riximyo	Rituximab	All indication	June 2017
17	Blitzima	Rituximab	All indications	July 2017
18	Ritemvia	Rituximab	All indications	July 2017
19	Rituzena	Rituximab	All indications	July 2017
20	Ontruzant	Trastuzumab	All indications	November 2017
21	Mvasi	Bevacizumab	All indications	January 2018
22	Herzuma	Trastuzumab	All indications	February2018
23	Kanjiniti	Trastuzumab	All indications	May 2018
24	Trazimera	Trastuzumab	All indications	July 2018
25	Pelgraz	Pegfilgrastim	Neutropenia	July 2018
26	Fulphila	Pegfilgrastim	Neutropenia	September 2018
27	Ratiograstim	Filgrastim	Neutropenia, HSCT	September 2018
28	Udenyca	Pegfilgrastim	Neutropenia	September 2018
29	Ogivri	Trastuzumab	All indications	October 2018
30	Pelmeg	Pegfilgrastim	Neutropenia	November 2018
31	Ziextenzo	Pegfilgrastim	Neutropenia	November 2018

EMA = European Medicines Agency, VTE = venous thromboembolism, HSCT = hematopoietic stem cell transplantation

**Table 2:** List of US FDA-approved oncology biosimilars, purple book[13]

**No.**	**Brand Name**	**Active substance**	**Indication**	**Authorization date**
1	Zarxio	Filgrastim-sndz	Neutropenia	March 2015
2	Mvasi	Bevacizumab-awwb	All indications	September 2017
3	Ogivri	Trastuzumab-dkst	All indications	December 2017
4	Retacrit	Epoetin Alfa-epbx	Anemia	May 2018
5	Fulphila	Pegfilgrastim-jmdb	Neutropenia	June 2018
6	Nivestim	Filgrastim-aafi	Neutropenia	July 2018
7	Udenyca	Pegfilgrastim-cbqv	Neutropenia	November 2018
8	Truxima	Rituximab-abbs	All indications	November 2018
9	Herzuma	Trastuzumab-pkrb	All indications	December 2018
10	Ontruzant	Trastuzumab-dttb	All indications	January 2019
11	Trazimera	Trastuzumab-qyyp	All indications	March 2019
12	Kanjinti	Ttrastuzumab-anns	All indications	June 2019
13	Zirabev	Bevacizumab-bvzr	All indications	June 2019

US FDA = United States Food and Drug Administration

**Table 3:** List of SFDA-approved oncology biosimilar agents[14]

**No.**	**Brand Name**	**Active substance**	**Indications**
1	Zarzio	Filgrastim 300 mcg and 480 mcg	Neutropenia
2	Gastrofil	Filgrastim 300 mcg and 480 mcg	Neutropenia
3	Binocrit	Epoetin Alfa 1000 U, 2000 U, 4000 U, 10,000 U, 20,000 U, 30,000 U, and 40,000 U	Anemia
4	Nivestim	Filgrastim 600 mcg and filgrastim 600 mcg	Neutropenia

SFDA = Saudi Food and Drug Authority

This study describes the pharmacoeconomic impact of biosimilar in oncology and formulary consideration of oncology biosimilars substituting with their originators in major oncology centers in Kingdom of Saudi Arabia (KSA). It also delineates challenges faced by biosimilars because of the approval of second-generation biologicals and how to address these challenges. This study also emphasizes on the need to have a rigorous pharmacovigilance efforts and naming strategies. The Ministry of National Guard Health Affairs (MNGHA) has recommended specific naming strategies of the biosimilars for effective pharmacovigilance monitoring of biologics and biosimilars.

## Pharmacovigilance of Biosimilars in Oncology

There are still some concerns regarding the long-term evaluation of biosimilars, particularly, the limited experience with these products in terms of efficacy, safety, and immunogenicity at the time of approval. For this reason, pharmacovigilance should be rigorous and is important as a public health concern. Ultimately, only clinical trials and effective post-marketing pharmacovigilance will provide definitive evidence that a biosimilar is comparable with the reference product in term of efficacy and safety. The aim of clinical trials with trastuzumab biosimilars was to show equivalence, and not patient benefit, as this was shown with brand trastuzumab.[15] Now biosimilars of trastuzumab, rituximab, and bevacizumab have been approved and we have several challenging issues that need to be addressed, such as maintaining appropriate pharmacovigilance, extrapolating across indications and automatic substitution, and switching. No consensus has yet been reached in any of these areas.

Clinical testing preapproval may not identify all possible adverse events (AEs) with most biologics, including biosimilars. An evaluation of clinical safety therefore is continued in the post-marketing setting. WHO guidance provides recommendations for post-marketing safety reports for product tolerability, and such reports include a scientific evaluation of frequency/causality of AEs. WHO also recommends that following approval, the manufacturers have a system in place to detect and assess, understand, and prevent any potentially drug-related AEs. This system, referred to as pharmacovigilance, also provides for notification regarding the occurrence of such AEs in whatever countries the product may be marketed. The goal of a post approval pharmacovigilance plan is to identify and understand, as fully as possible, the frequency and nature of AEs associated with a specific product, including potential risk factors for such AEs.[11]

To address safety considerations, the EMA mandates post approval monitoring, as well as pharmacovigilance plans for biologic drugs, including biosimilars.[16] In addition, WHO and EMA recommend that if, based on clinical experience, any additional specific safety monitoring or pharmacovigilance plan has been required for the reference biologic, or its specific product class (e.g., erythropoietin stimulating agents), the same plan should be applied to the biosimilar. Likewise, if additional concerns (e.g., increased immunogenicity of the biosimilar) have arisen during the evaluation of the biosimilar product, these also may be evaluated through appropriate safety monitoring.[12]

US FDA guidance on Good Pharmacovigilance Practice considers routine spontaneous AEs reporting to be sufficient post-marketing surveillance for products where no safety risks have been identified pre- or postapproval, and if used in adequately studied populations. US FDA considers a specific pharmacovigilance plan as appropriate; however, in the event the at-risk population needs additional study, or if safety risks have been identified either pre- or postapproval. As defined by existing US FDA guidance, such a pharmacovigilance plan could include additional measures beyond routine reporting, such as expedited reporting of serious AEs, active surveillance for specific AEs, creation of product registries, pharmacoepidemiologic studies, or additional clinical trials.[20]

Nomenclature and Product Labeling Considerations Naming is an important consideration when developing regulatory policies for biosimilars because of its potential impact on physician prescribing or patient bias, interchangeability, as well as pharmacovigilance. It is important that biosimilars have names that make them readily distinguishable from the innovator biologic (as well as other biosimilar products).[17,18] This is necessary to make certain that AEs that occur in the post-market setting can be readily and correctly matched to a specific product.[17,19] US FDA has published Nonproprietary Naming of Biological Products guidance for industry in January 2017. With the introduction of more biological products, US FDA believes it is important to encourage routine use of designated suffixes in ordering, prescribing, dispensing, recordkeeping, and pharmacovigilance practices for biological products, irrespective of their licensure pathway and date of licensure. The designated suffix will provide a consistent, readily available, and recognizable mechanism for patients and healthcare professionals, including providers and pharmacists, to correctly identify these products. US FDA believes it is likely that US FDA-designated suffixes will be used routinely when identifying, describing, and recording use of biological products if such suffixes are present in the proper names of all biological products licensed under the Public Health Service (PHS) Act.[20]

Some position statements suggest the International Nonproprietary Name (INN) system should not be used to prescribe biologic drugs.[21] One of the reasons for this is that INN nomenclature with biosimilars can lead to problems, for example, if some countries allow pharmacists to auto-substitute a less-expensive drug having the same INN as its reference product.[22] Instead, naming according to product brand has been recommended to enable better pharmacovigilance of biosimilars, so specific events can be associated with the correct product and manufacturer.[19,21]

Corporate Pharmacy and Therapeutic committee at MNGHA has approved a naming strategies policy for biosimilars in MNGHA formulary. It has recommended to use brand names to be included in computerized prescribing order entry in Health Information System in addition to the generic name (INN) of the drug to allow tracking for pharmacovigilance monitoring. The biosimilar product is identified as a “biosimilar” in the order entry screen by adding the term (Biosimilar) to the product's name. Biosimilars have a different formulary codes than the reference product. Other hospitals in the country can also use the same naming strategy for effective pharmacovigilance monitoring.

Healthcare practitioners in KSA should be encouraged to engage effectively in pharmacovigilance efforts and monitor and report AEs, efficacy concerns, immunogenicity concerns, and medication errors associated with biosimilars to SFDA. Healthcare practitioners should document correct attribution of safety event, for example, what was ordered vs. what did the patient receive in maintenance of electronic medical record, bar code administration, and medication reconciliation (during transition of care).

### Pharmacoeconomic impact of biosimilars in oncology and formulary consideration of oncology biosimilars

Biologics are significant drivers of globally escalating healthcare costs.[23] Biologic agents play an increasingly important role in the medical care of patients. Between 2010 and 2015, biologics accounted for 22% of drugs approved by the US FDA. By 2019, it is estimated that the global market for biologics will reach $66.4 billion.[24] Therefore, there is a need to integrate biosimilars into oncology practice in order to reduce medication costs to manage healthcare resources responsibly and expand the access of these biologics to more cancer patients.

Biosimilars have potential to offer cost savings with comparable efficacy and safety to innovator products and increase the access of treatment to more patients. Analytic tools to determine pharmacoeconomic value of biosimilar are opportunity cost, cost-effectiveness, and cost-minimization analyses (CMAs). CMAs should be used when comparing one biosimilar to originator considering they have same efficacy and safety.[23] CMA compares the cost of two similar interventions to ascertain which is less expensive. However, it is limited by the fact that it often compares two different interventions that may initially seem similar but are not. If there are differences in the effectiveness of a biosimilar and the comparator, other techniques of economic evaluation need to be used, such as cost-effectiveness analysis or cost-utility analysis.[25]

The gross domestic product (GDP) measures the value of economic activity within a country. Strictly defined, GDP is the sum of the market values, or prices, of all final goods and services produced in an economy during a period of time. GDP Purchasing Power Parity (GDP PPP) of United Kingdom (UK), Canada, and KSA were $41,602, $35,256, and $21,395 USD, respectively, in 2016. Threshold incremental cost-effectiveness ratio (ICER)/quality-adjusted life-year (QALY) for drug approvals in UK is $26,157 to $39,236 USD. However, the threshold ICER/QALY for drug approvals in Canada is $38,000 USD. It appears that threshold ICER/QALY for drug approvals in UK and Canada is close to GDP PPP in their respective countries. We do not have threshold ICER/QALY as a benchmark for drug approvals in the KSA yet. However, regulatory authority, SFDA, has approved many cancer drugs in the country, which are costing more than two to three times of GDP PPP of KSA using the direct cost of the drugs (excluding the hidden cost of best supportive care and others). Hence, it is imperative to establish threshold ICER/QALY benchmark for drug approvals in the KSA. Until, we come up with local benchmark threshold ICER, we need to develop strategies to lower the cost of oncology medications in the country.

### Anticipated cost impact of approval of biosimilar trastuzumab

Breast cancer is the most common cancer as per Saudi Cancer registry 2015 with total number of newly diagnosed breast cancer cases of 2016.[26] Around 25% breast cancer patients are human epidermal growth factor receptor 2 (her2neu) positive and need trastuzumab. Cost of one patient receiving trastuzumab per year is 125,062 Saudi Arabian Riyal (SAR) ($33,350 USD) when used alone. Yearly cost is 62.5 million SAR ($16.7 million) for 500 patients. Expected saving by approving biosimilar trastuzumab in the KSA will be nearly 30 million SAR (8 million). Current standard of care adjuvant therapy for her2neu positive breast cancer in our hospital is chemotherapy plus trastuzumab which costs 146,952 SAR ($39,187) per patient. US FDA and National Comprehensive Cancer Network (NCCN) guidelines have also approved a combination of chemotherapy, trastuzumab, and pertuzumab as adjuvant therapy for her2neu positive breast cancer based on APHINITY Trial.[27] APHINITY was a Phase-III randomized controlled clinical trial which compared combination of chemotherapy, trastuzumab, and pertuzumab q3 weeks vs. Chemotherapy and trastuzumab in the adjuvant setting for the management of her2neu positive breast cancer patients. After a median follow-up of 45.4 months, the proportion of invasive disease-free survival (iDFS) events in the intent-to-treat population was 7.1% (*n* = 171) in the pertuzumab arm and 8.7% (*n* = 210) for those receiving placebo (HR 0.82; 95% CI: 0.67, 1.00; *P* = 0.047). However, cost per patient per treatment course is 427,104 SAR ($113,894) when combination of chemotherapy, trastuzumab, and pertuzumab is used. It is not cost effective and hence this indication was rejected by MNGHA as its direct cost is 5.5-fold higher than GDP PPP in KSA. Cost of trastuzumab plus chemotherapy combination is 146,952 SAR ($39,187) per patient when used in adjuvant setting. It is more cost-effective as compared to combination of trastuzumab, pertuzumab, and chemotherapy in the adjuvant setting but it still costs two-fold higher than GDP PPP of KSA. Threshold for approval of drugs should be close to GDP PPP of the country as is witnessed in many countries including UK and Canada, which is 78,000 SAR ($21,000) in the KSA if we compare ourselves with UK model. Substitution of brand trastuzumab with biosimilar trastuzumab will result in saving of 62,500 SAR ($16,667) per patient. Cost of the adjuvant regimen per patient is expected to be 83,500 SAR ($22,266) when brand trastuzumab will be substituted with biosimilar trastuzumab which is close to GDP PPP.

### Anticipated cost impact of approval of biosimilar trastuzumab, biosimilar rituximab, and biosimilar bevacizumab at MNGHA, Saudi Arabia

Annual oncology medications expenditures have been increasing by 10% every year for the past few years at MNGHA. Oncology formulary medication expenditures in 2016 were 171.6 million SAR (45.76 million) in MNGHA. Trastuzumab, rituximab, and bevacizumab were among the top 10 most expensive oncology medications at MNGHA with expenditures of 20.78 million SAR (5.55 million), 18.07 million SAR (4.82 million), and 11.24 million SAR (3 million), respectively. Total expenditures of these three monoclonal antibodies in 2016 were 50 million SAR (13.33 million) which accounted for 29% of the annual formulary medication expenditures in 2016. Oncology medication expenditures at MNGHA have been expected to have been increased by around 30% in 2019. Estimated expenditures of aforementioned three expensive biologicals will be 65 million SAR approximately in 2019 (17.33 million) at MNGHA. There will be significant cost-saving impact of adding biosimilars of three biologics (trastuzumab, rituximab, and bevacizumab) in MNGHA formulary. It is estimated that originator will reduce the price by around 30% on the approval of biosimilars and consequently biosimilar companies will reduce their cost by another 30% in order to be competitive with the innovators. Hence, it is anticipated to have around 60% savings by substituting the brands of rituximab, trastuzumab, and bevacizumab with their approved biosimilars at MNGHA which corresponds to 39 million SAR (10.4 million) saving annually.

### Cost impact of addition of biosimilar GCSF in the formulary: local experience of biosimilar filgrastim use at MNGHA, Saudi Arabia

We would like to share practical example of filgrastim biosimilar use in MNGHA. We have three different granulocyte colony-stimulating factors (GCSF) in MNGHA formulary, filgrastim 300 mcg/mL (Neupogen), biosimilar filgrastim 300 mcg/0.5 mL (Zarzio), and Pegfilgrastim (Neulasta) [Table 4].

**Table 4:** Cost impact of addition of biosimilar GCSF in the formulary

	**Unit price, SAR (USD)**	**Annual Consumption in MNGHA, injections**	**Annual Cost**
Filgrastim 300 mcg/mL (Neupogen)	225 (60)	7898 (23%)	1.77 million (472,000)
Biosimilar Filgrastim 300 mcg/0.5 mL (Zarzio)	96 (25.6)	26,908 (77%)	2.58 million (688,000)
Total		34,806 Injections	4.36 million (1.16 million)

	**Total annual price, SAR (USD)**	**Total Annual Cost Savings of Using Biosimilar GCSF**	**Total Annual Cost Savings of Adding Biosimilar Filgrastim to Formulary**

Cost of brand Neupogen using current price of 225 SAR/($60) injection	7.83 million (2.08 million)	3.47 million (925,333)	20 million (5.33 million)
Cost of brand Neupogen using previous price of 450	15.66 million	11.30 million (3 million)	60 million (16 million)
SAR ($120)/injection before the addition of biosimilar GCSF	(4.17 million)		

Note - Values inside parentheses are USD unless otherwise noted. GCSF = Granulocyte colony-stimulating factors, MNGHA = Ministry of National Guard Health Affairs, SAR = Saudi Arabian Riyal, USD = United States Dollar.

We use GCSF as a primary prophylaxis based on the American Society of Clinical Oncology (ASCO) 2006 guidelines which recommends use of GCSF when the risk of febrile neutropenia (FN) is in the range of 20% or higher in cancer patients receiving chemotherapy. GCSF is also recommended in patients who have a high risk of FN regardless of the incidence of FN. There is evidence for the use of GCSF for the management of established FN. Adding GCSF to antibiotic therapy does not improve survival but it shortens the duration of neutropenia, reduces the duration of antibiotic therapy and hospitalization, and decreases hospital costs in patients with high-risk FN.[28] Moreover, biosimilar filgrastim has also been used successfully for mobilization of stem cells in both allogenic and autologous HSCT patients. Corporate Pharmacy and Therapeutic Committee added biosimilar filgrastim (Zarzio) into MNGHA formulary in February 2014.

Price of Neupogen brand was 450 SAR ($120) per vial before addition of Zarzio which was reduced to 225 SAR ($60) after biosimilar was added into MNGHA formulary. Price of biosimilar filgrastim has been set at 96 SAR ($25.6) per vial at MNGHA. We used a total of 34,806 injections of GCSF during the period August 2017 till July 2018 in MNGHA. Consumption of biosimilar GCSF was 26,908 injections during the said period, which is representing 77% of the total consumption costing 2.58 million SAR ($688,000) whereas consumption of filgrastim (Neupogen) was 7,898 injections, representing 23% of the total consumption costing 1.77 million SAR ($472,000). Total expenditures of the 34,806 injections used during the said period was 4.36 million SAR (1.16 million). Seventy seven percent conversion of brand neupogen to biosimilar filgrastim resulted in saving of 3.47 million SAR ($925,333) per year using the current price of 225 SAR ($60) per vial. If we compare the cost of biosimilar filgrastim with historical price of brand filgrastim (neupogen) of 450 SAR ($120) per injection, then the annual saving is 11.30 million SAR per year (3 million). It has been more than 5 years as we started using biosimilar filgrastim. Hence, if we extrapolate this saving over past 5 years and half, then we have saved around 60 million SAR (16 million) by adding biosimilar filgrastim to the MNGHA formulary. This is a significant cost saving on the stretched budget of MNGHA, and this saving can be used to expand cancer care services at MNGHA [Table 4].

### Challenges faced with biosimilars due to approval of second-generation biologicals

The emergence of second-generation biologics (or biologics that make improvements on existing biologics through pegylation, alternative formulations, or other means) may affect the value of not only first-generation reference biologics, but also their biosimilars. As research and development of biologics in the oncology setting continues, newer, second-generation biologic drugs may offer different clinical properties compared with currently approved reference biologics.[23,29] They may include new formulations, different efficacy profiles and/or dosing regimens, or reduced immunogenicity. A second-generation biologic may have an improved efficacy and/or safety profile, but if the efficacy and safety of a given second-generation drug is comparable to the first-generation drug or its biosimilar, a CMA could be performed to identify the most economical solution for patients and payers. In contrast, cost-effectiveness comparison analyses could be performed with novel biologics that have different efficacy and/or safety profiles relative to first-generation products or their biosimilars. The results of pharmacoeconomic analyses that incorporate second-generation biologic drugs may affect the value that biosimilars of first-generation reference biologic drugs offer patients with cancer and healthcare providers. This may include the extent of financial and opportunity costs offset by the emergence of these therapies. For example, if a second-generation biologic has improved efficacy, the opportunity for the patient to have a better outcome would possibly negate its higher cost. In addition, the emergence of second-generation biologic drugs may affect the drug acquisition prices for first-generation reference biologic drugs and their biosimilars.[23] Manufacturers of second-generation biologics have extended their patency for another 15–20 years.

Rituximab (IV Injection's patency was expired in 2018 but the pharmaceutical company manufacturing rituximab has developed second-generation rituximab in the form of subcutaneous (SC) dosage form. EMA approved the SC rituximab in 2014 and later on US FDA has also approved it in 2017. There is a major advantage with SC rituximab with regards to ease and convenience of its administration pattern. SC rituximab is a fixed dose of 1400 mg or 1600 mg and is administered as SC over 5 min as opposed to IV over more than 6 h. It seems that extent of financial and opportunity costs offered by biosimilar rituximab are offset by the emergence of second-generation SC rituximab. Trastuzumab IV Injection's patency has already been expired in USA in June 2019. EMA has approved SC trastuzumab in 2013. US FDA has also approved SC trastuzumab and it is already approved by SFDA. Advantage of second-generation trastuzumab is a fixed dose of 600 mg which is administered as SC injection as opposed to IV administration over 0.5–1.5 h. It appears that extent of financial and opportunity costs offered by biosimilar trastuzumab are offset to a certain degree by the emergence of second-generation trastuzumab (SC). However, the difference in the administration time between IV and SC trastuzumab dosage form is not significant as have been seen in the case of SC rituximab vs. IV rituximab. Bevacizumab IV Injection's patency will expire in 2020 and SC bevacizumab is in Phase-II Clinical Trials. In our opinion, it seems difficult to substitute SC rituximab with biosimilar rituximab because of its convenient administration pattern saving significant amount of infusion center time. However, intravenous (IV) and SC trastuzumab can be substituted with IV biosimilar trastuzumab because the difference in the administration time between IV and SC trastuzumab dosage form is not significant and this substitution will have huge impact on cost saving. Similarly, IV bevacizumab can be substituted with its biosimilar bevacizumab resulting in significant cost saving.

## Summary and Conclusion

Biosimilar is a biologic product that is highly similar to the reference product, notwithstanding minor differences in clinically inactive components. There are no clinically meaningful differences between the biosimilar and the reference product in terms of safety, purity, and potency.

Corporate Pharmacy and Therapeutic committee at MNGHA has approved a naming strategies policy for biosimilars in MNGHA formulary. It has recommended to use brand names to be included in computerized prescribing order entry in Health Information System in addition to the generic name (INN) of the drug to allow tracking for pharmacovigilance monitoring. Other hospitals in the country can also use the same naming strategy for effective pharmacovigilance monitoring. Healthcare practitioners in KSA should be encouraged to engage effectively in pharmacovigilance efforts when using biologicals including the biosimilars and monitor and report AEs, efficacy concerns, immunogenicity concerns, and medication errors to SFDA.

Biologics are significant drivers of globally escalating healthcare costs. Biosimilars have potential to offer cost savings with comparable efficacy and safety to innovator products and increase the access of treatment to more patients. Substitution of filgrastim (neupogen brand) with biosimilar filgrastim has resulted in significant cost saving when biosimilar filgrastim was used for prophylaxis and management of FN as well as for mobilization of stem cells in MNGHA. Moreover, substitution of three commonly used monoclonal antibodies such as rituximab, trastuzumab, and bevacizumab with their approved biosimilars will have significant cost saving in different oncology hospitals of the KSA.

Second-generation biologics may have improved, efficacy, tolerability, and convenient administration pattern saving infusion center time. In our opinion, it seems difficult to substitute SC rituximab with biosimilar rituximab because of convenient administration pattern saving infusion center time. However, IV and SC trastuzumab can be substituted with IV biosimilar trastuzumab because the difference in the administration time between IV and SC trastuzumab dosage form is not significant and this substitution will have huge impact on cost saving. Similarly, IV bevacizumab can be substituted with its biosimilar bevacizumab resulting in significant cost saving.
